# Genome-Wide Association Study and Cost-Efficient Genomic Predictions for Growth and Fillet Yield in Nile Tilapia (*Oreochromis niloticus*)

**DOI:** 10.1534/g3.119.400116

**Published:** 2019-06-06

**Authors:** Grazyella M. Yoshida, Jean P. Lhorente, Katharina Correa, Jose Soto, Diego Salas, José M. Yáñez

**Affiliations:** * Facultad de Ciencias Veterinarias y Pecuarias, Universidad de Chile, Santiago, 8820808 Chile,; † Benchmark Genetics Chile, Puerto Montt, Chile, and; ‡ Grupo Acuacorporacion Internacional (GACI), Cañas, Costa Rica

**Keywords:** complex traits, cost-efficient, genomic prediction, GWAS, genotype imputation, low-density panel, *Oreochromis niloticus*, GenPred, Shared Data Resources

## Abstract

Fillet yield (FY) and harvest weight (HW) are economically important traits in Nile tilapia production. Genetic improvement of these traits, especially for FY, are lacking, due to the absence of efficient methods to measure the traits without sacrificing fish and the use of information from relatives to selection. However, genomic information could be used by genomic selection to improve traits that are difficult to measure directly in selection candidates, as in the case of FY. The objectives of this study were: (i) to perform genome-wide association studies (GWAS) to dissect the genetic architecture of FY and HW, (ii) to evaluate the accuracy of genotype imputation and (iii) to assess the accuracy of genomic selection using true and imputed low-density (LD) single nucleotide polymorphism (SNP) panels to determine a cost-effective strategy for practical implementation of genomic information in tilapia breeding programs. The data set consisted of 5,866 phenotyped animals and 1,238 genotyped animals (108 parents and 1,130 offspring) using a 50K SNP panel. The GWAS were performed using all genotyped and phenotyped animals. The genotyped imputation was performed from LD panels (LD0.5K, LD1K and LD3K) to high-density panel (HD), using information from parents and 20% of offspring in the reference set and the remaining 80% in the validation set. In addition, we tested the accuracy of genomic selection using true and imputed genotypes comparing the accuracy obtained from pedigree-based best linear unbiased prediction (PBLUP) and genomic predictions. The results from GWAS supports evidence of the polygenic nature of FY and HW. The accuracy of imputation ranged from 0.90 to 0.98 for LD0.5K and LD3K, respectively. The accuracy of genomic prediction outperformed the estimated breeding value from PBLUP. The use of imputation for genomic selection resulted in an increased relative accuracy independent of the trait and LD panel analyzed. The present results suggest that genotype imputation could be a cost-effective strategy for genomic selection in Nile tilapia breeding programs.

Investment in selective breeding programs generates economic return because genetic selection is aimed at improving productivity of important economic traits that result in permanent, cumulative and sustainable changes in a farm’s population over generations of selection (Gjedrem 2012). In a simulation study, Ponzoni *et al.* (2007) estimated that the benefit/cost ratio reached a maximum of 60/1 with the implementation of family based breeding programs in Nile tilapia. The improvement in the benefit/cost ratio by using genomic information has never been reported in the literature for aquaculture species. However, Sonesson *et al.* (2009) suggested that the extra cost of genotyping can be partly recovered by higher genetic gains due to the increased accuracy by genomic prediction compared to breeding values estimated using conventional pedigree-based best linear unbiased prediction (BLUP). Therefore, selective breeding is an important tool to increase aquaculture production and profitability, satisfying the increasing demand for animal protein (Gjedrem 2012).

The first Nile tilapia breeding program was established in 1988 and since then high levels of genetic gains have been achieved for economically important traits, *e.g.*, genetic gains for body weight ranged from 7 to 20% per generation (Bentsen *et al.*, 2017; Eknath *et al.*, 1998; Gjedrem *et al.*, 2012 and Khaw *et al.*, 2008). However, until now the Nile tilapia breeding programs have been based only on pedigree and phenotype information for genetic evaluations. The incorporation of genomic information for genetic analysis has not been evaluated or implemented in tilapia breeding programs. This is mainly due to the fact that dense SNP panels were not available until recently (Joshi *et al.* 2018; Yáñez *et al.* 2019). The use of genomic information for the implementation of genomic selection has already been assessed in various aquaculture species, *e.g.*, Atlantic salmon, rainbow trout, salmon coho, common carp, channel catfish and Pacific oyster (Barria *et al.*, 2018; Garcia *et al.*, 2018; Gutierrez *et al.*, 2018; Palaiokostas *et al.*, 2018; Vallejo *et al.*, 2018; Yoshida *et al.*, 2018a; Bangera *et al.*, 2017; Correa *et al.*, 2017; Meuwissen *et al.*, 2014; Ødegård *et al.*, 2014; Tsai *et al.*, 2016). As it has been demonstrated in these studies, an increase in selection accuracy when including genomic information from dense SNP panels, especially for traits which are difficult to measure in selection candidates (Yañez and Martinez 2010; Yáñez *et al.* 2014). Carcass quality traits (*e.g.*, fillet yield) are considered key traits in the breeding goal for Nile tilapia genetic improvement (Nguyen *et al.* 2010; Ponzoni *et al.* 2011) and these traits could be more efficiently improved through the inclusion of genomic information in genetic evaluations.

The use of genomic information from dense SNP panels provides the opportunity to increase the rate of genetic progress in breeding programs (Meuwissen *et al.* 2001). However, the cost of genotyping is high and alternative methods are necessary for cost-efficient genomic applications (VanRaden, *et al.* 2011; Carvalheiro *et al.* 2014). Strategies such as selective genotyping (Sen *et al.* 2009; Jiménez-Montero *et al.* 2012; Ødegård and Meuwissen 2014), genotyping animals using low-density panels (Tsai *et al.* 2016; Bangera *et al.* 2017; Correa *et al.* 2017; Yoshida *et al.* 2018a) and genotype imputation (Cleveland and Hickey 2014; Sargolzaei *et al.* 2014; Chen *et al.* 2014) have been tested as alternative strategies for reducing costs for the practical implementation of genomic information in breeding programs.

Imputation of genotypes reduces the cost of genomic selection by genotyping a small proportion of animals (*e.g.*, parents or influential animals) using a dense SNP panel and selection candidates using a LD SNP panel, and then imputing (predicting) missing genotypes from the lower to the HD SNP panel (Sargolzaei *et al.* 2009). In aquaculture species, these cost-effective strategies have been assessed and reported to generate genomic prediction accuracies similar to those obtained when all selection candidates are genotyped with HD SNP panels (Dufflocq *et al.*, 2019; Tsai *et al.*, 2017; Yoshida *et al.*, 2018b).

The objectives of this study were: (i) to perform a genome-wide association study to dissect the genetic architecture and identify molecular markers for growth and fillet yield; (ii) to evaluate the accuracy of genotype imputation as a cost-effective strategy for genotyping, and (iii) to assess the accuracy of genomic selection for growth and fillet yield using true and imputed SNP genotypes in farmed Nile tilapia. To our knowledge, this is the first study evaluating the incorporation of true and imputed dense genotypes for the implementation of genomic predictions in farmed Nile tilapia.

## Material And Methods

### Phenotypes

The Nile tilapia population used in the current study belong to a breeding nucleus established by Aquacorporación Internacional group (GACI) in Costa Rica. The origin of the population is described in detail by Yoshida *et al.* (2019a). This population consisted of eight generations selected for growth rate. Here, we used phenotype information for fillet yield and harvest weight from four generations. In addition, for all analysis (GWAs, genotype imputation and genomic predictions) we used the pedigree information of all animals from the eight generations (65,570 animals). To generate the families from each year-class, briefly, the eggs of each full-sib family were incubated and reared in separate hapas until tagging. A mating design of two dams per sire was used to produce full and half-families, which varied from 74 to 89 families across the year-classes (Table 1). For each year-class an average number of 18 fish/family (ranging from 5 to 49) were tagged at an average weight and age of 13 g (SD = 8 g) and 104 days (SD = 18 days), respectively. After, the fish were reared until an average of 13 months old, where the traits fillet yield (FY (%) = (fillet weight/harvest weight*100) and harvest weight (HW in grams) were recorded for each individual fish and the fillet weight was measured for both fillet.

**Table 1:** Summary statistics for phenotyped animals by year-class

Year-class	Number of Families	Animals genotyped	Age		Fillet Yield			Harvest Weight		
			Mean	SD^2^	Number	Mean (%)	SD^2^	Number	Mean (g)	SD^2^
**2011**	89	—	376.25	24.24	1,004	36.34	1.85	1,027	919.15	257.61
**2012**	82	—	343.48	16.33	0760	34.47	2.05	0767	730.79	235.62
**2013**	80	—	514.22	14.49	2,628	34.07	2.45	2,636	907.91	268.04
**2017**	74	1,130*	370.54	20.04	1,474	31.74	2.16	1,479	953.57	252.86

* Number of genotyped animals after quality control. Additionally 108 parents of year-class 2017 were genotyped using 50K SNPs panel to perform genotype imputation.

### Genotypes

Genomic DNA was extracted from fin clip samples from 108 parents (45 sires and 63 dams) and 1,364 offspring from year-class 2017. Samples were then genotyped using a 50K SNP Illumina BeadChip, which is described in detail by Yáñez *et al.* (2019). Genomic DNA was purified from all the samples using the DNeasy Blood & Tissue Kit (QIAGEN) according to the manufacturer’s protocol. Before the genome-wide association study (GWAS) and imputation analysis, genotypes and samples were filtered according to the following exclusion criteria: Hardy-Weinberg Disequilibrium (HWE, p-value < 1×10^−6^), Minor Allele Frequency (MAF < 0.05) and genotyping call-rate for SNP and samples < 0.95.

### Genome-wide association analysis

We performed the GWAS to dissect the genetic architecture and to identify regions of the Nile tilapia genome containing SNPs with important effects on FY and HW. We used the weighted single step genomic best linear unbiased prediction (wssGBLUP) method (Wang *et al.* 2012) implemented in postGSf90 module from BLUPf90 family programs (Misztal *et al.* 2016). The following model was used:

y=Xβ+Za+Wc+e (1)

where y is a vector of phenotypes (FY or HW), β is a vector of contemporary group as fixed effects that comprise the year-class:sex:tank, and harvest weight or age for FY and HW as covariate, respectively; a is a vector of random additive direct genetic effects; c is a vector of common environmental effect and e is a vector of residual effect. X, Z and W are incidence matrices for β, a and c effects, respectively.

The wssGBLUP is similar to the pedigree-based BLUP (PBLUP) method except for the use of a combined genomic and pedigree relationship. The kinship matrix A^−1^ is replaced by matrix H^−1^ (Aguilar *et al.* 2010), which combines genotype and pedigree relationship coefficients:

$$H^{-1}=A^{-1}+\left[\begin{array}{cc} 0 & 0\\ 0 & G^{-1}-A_{22}^{-1} \end{array}\right],$$

where, $A_{22}^{-1}$ is the inverse of a pedigree-based relationship matrix for genotyped animals; and $G^{-1}$ is the inverse genomic relationship matrix. The SNPs were assumed with an initial value of one corresponding to the single-step genomic BLUP (ssGBLUP) method (Misztal *et al.* 2009). In the wssGBLUP the marker variances were estimated from allele frequencies and used as weights, which were updated on each iteration (Wang *et al.* 2014). We tested three iterations of weights, and used the second iteration to manhattans plots, as suggested by Wang *et al.* (2012) and Zhang *et al.* (2016), who proposed that two iterations were sufficient to correctly identify major SNPs in wssGBLUP. The wssGBLUP included all HD genotyped animals (parents and offspring) which passed quality control (n = 1,238) and all the phenotyped fish present in Table 1.

To evaluate the presence of putative genes associated with the traits under study, we reported all genes between the first and last SNP position of each 20-SNP window, searched using BLAST (*Basic Local Alignment Search Tool*) of the SNP probes against the last version of the *Oreochromis niloticus* reference genome (Conte *et al.* 2019), publicly available at NCBI (GenBank assembly accession GCA_001858045.3).

### Genotype imputation

Three *in silico* LD panels were constructed with SNP densities of 500 (LD0.5K), 1,000 (LD1K) and 3,000 (LD3K). The SNPs from the LD panels were selected using the option–indep-pairwise of Plink v1.9 software (Purcell *et al.* 2007), with a window size of 180,432 kb, a step of 1 SNPs and a variable r^2^ according to chromosome. This command produced a subset of markers that are proportional for chromosome size, are approximately evenly spaced and in low linkage disequilibrium with each other as recommended by Cleveland and Hickey (2014).

The imputations were run using genotype information from 108 parents and 20% of the offspring (n = 225) as a reference set (HD panel) and 80% of offspring (n = 904) were used as the validation set (LD panel). The assignment of the offspring to the reference and validation sets was random, and five replicates were used each time. In addition, we used pedigree information available for all individuals for imputation (65,770 animals), consisting of eight generations of selection. Imputation of genotypes was performed using the FImpute v2.2 software (Sargolzaei *et al.*, 2014) and the accuracy of imputation was calculated as the correlation between true and imputed genotypes for the validation set.

### Genomic prediction

We used two scenarios to assess prediction accuracies using a fivefold cross validation. The first scenario used the true genotyped LD panels (LD0.5K, LD1K and LD3K) and represented the same *in silico* LD panel constructed for genotype imputation. The second scenario used the imputed genotypes from LD0.5K, LD1K and LD3K to 32K panel. In addition, accuracy of breeding values was also estimated using pedigree-based information (PBLUP method) and the true 32K SNPs (true markers that passed in quality control). Briefly, all genotyped animals (n = 904) with phenotypes were randomly divided into five exclusive training sets (80% of the dataset; n = 721 and SD = 5 animals) which were used to estimate the SNP effects; the remaining animals were used as validation sets (20% of the dataset; mean = 193 and SD = 5 animals), for which their phenotypes were masked and their performance was predicted based on the marker effects. This fivefold cross validation was replicated five times for each SNP panel density and the results are presented as a mean for all replications.

We used the BLUPF90 family of programs (Misztal *et al.* 2016) to perform the genetic evaluations using pedigree-based information and the ssGBLUP method which uses both pedigree and genomic information, and additional information of the animals with only phenotypes (Table 1) in the validation set. The statistical model fitted was the same of the equation 1, except for PBLUP method, for which the kinship matrix used was A^−1^ instead of H^−1^ in ssGBLUP.

Prediction accuracies were calculated in the validation sets using the following equation:

$$r_{(G)EBV,BV}=\frac{r_{(G)EBV,y}}{h}$$ (2)

where $r_{GEBV,y}$ is the correlation between the estimated breeding value (EBV) or genomic estimated breeding values (GEBV) of a given model (predicted for the validation set using information from the training set) and the true phenotypic record, while h is the square root of the pedigree-based estimate of heritability.

### Genetic parameters and heritability

The total additive genetic variance ($\sigma_{a}^{2})$ was estimated using the kinship matrix A and H for PBLUP and ssGBLUP, respectively. For all traits studied, the heritabilities were computed using the following equation:

$$h^{2}=\frac{\sigma_{a}^{2}}{\sigma_{a}^{2}+\sigma_{c}^{2}+\sigma_{e}^{2}}$$ (3)

where, $\sigma_{c}^{2}\;and\;\sigma_{e}^{2}$ is the common environmental and residual variance, respectively.

### Cost evaluation

We evaluated the direct savings when genotyping a proportion of animals using a LD panel and performing genotyping imputation. Costs were calculated on the basis of one discrete generation, for seven different sizes of a Nile tilapia breeding populations, ranging from a total of 4,150 to 10,150 fishes, with half of the animals were used as reference population (RP) and the other half as selection candidates (SC). All animals descend from a fixed number of parents (P; 100 females and 50 males) (Table S2). We evaluated four different scenarios: scenario A: all animals (P, RP and SC) were genotyped using a HD panel; and, scenario B, C and D, in which all parents and 20% of RP were genotyped using a HD SNP panel and the remaining animals were genotyped using a 3K, 1K or 0.5K LD panel, respectively. The genotyping cost was calculated assuming prices of U$50, $25, $20 and $10 per sample for HD (50K), 3K, 1K, and 0.5K, respectively. In addition, for scenario A, we assumed a price reduction of 10% in each increase of 1,000 animals genotyped using a HD panel, which resulted in prices ranging from $50 to $26.60 per sample for genotyping 4,150 and 10,150 animals, respectively. The results of the genotyping cost evaluation (Table S3) are presented as terms of cost reduction (%) comparing scenario A to other scenarios.

### Data availability

All raw phenotypic and genotypic data used in the current study can be found at Figshare public repository (https://figshare.com/s/9b265a22b7e138c5a839). Furthermore, Supplementary Figures (Figure S1-S4) and Supplementary Tables (Table S1-S3) are available at Figshare: https://figshare.com/s/9b265a22b7e138c5a839.

## Results

### Basic statistics and genotype quality control

The total number of individuals phenotyped ranged from 5,866 to 5,909 for FY and HW, respectively, and varied per year-class with the maximum number of animals phenotyped in 2013. On average, the recorded fish were 401 days old at harvest weight. The average FY was 34.2% (SD = 2.13% g) and the average HW was 878 g (SD = 254 g) for phenotyped fish (Table 1).

Out of the initial 1,364 individuals and 43,272 SNPs which were effectively genotyped, a total of 1,130 animals and 32,306 SNPs (32K) passed in the quality control. The MAF < 0.05 parameter excluded the highest number of SNPs (4,779), whereas HWE and genotyped call-rate excluded 1,982 and 4,205 SNPs, respectively.

### Genetic parameters and heritability

For both FY and HW the additive genetic variance and heritability were slightly higher when using genomic information compared to the pedigree-based method. For instance, heritability values using ssGBLUP were 0.21 and 0.36 for FY and HW, respectively. For PBLUP heritability for FY and HW was estimated to be 0.21 to 0.31, respectively (Table 2). Additionally, a reduction in error of heritability estimates was shown for ssGBLUP when compared with PBLUP.

**Table 2:** Estimates of variance components and heritability for fillet yield and harvest weight in Nile tilapia

Traits	PBLUP					ssGBLUP*				
	$\sigma_{a}^{2}$^1^	$\sigma_{c}^{2}$^2^	$\sigma_{e}^{2}$^3^	h^2^^4^	SE^5^	$\sigma_{a}^{2}$^1^	$\sigma_{c}^{2}$^2^	$\sigma_{e}^{2}$^3^	h^2^^4^	SE^5^
**Fillet yield**	0.972	0.174	3.498	0.209	0.053	0.972	0.168	3.491	0.210	0.038
**Harvest weight**	19,312	4,296	39,522	0.306	0.073	23,161	3,823	37,345	0.360	0.047

* Estimated for true 32K genotype panel.

1 Additive genetic variance;

2 Common environment variance;

3 Residual variance;

4 Heritability;

5 Standard error.

### Genome-wide association analysis

**Figure 1:**
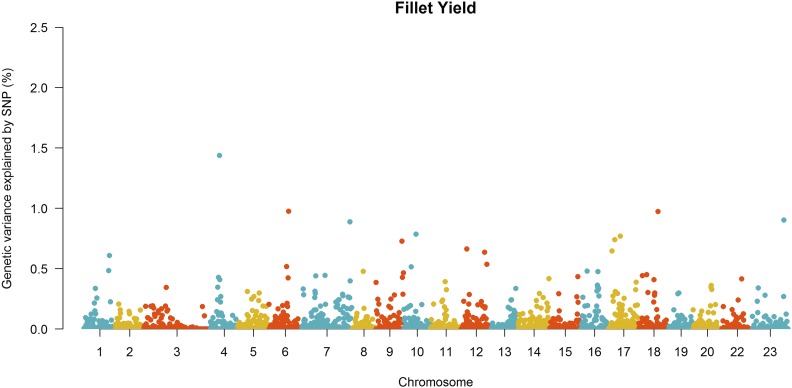
Manhattan plot of genetic variance explained by 20-SNP windows for fillet yield in the 2^nd^ iteration of wssGBLUP.

**Figure 2:**
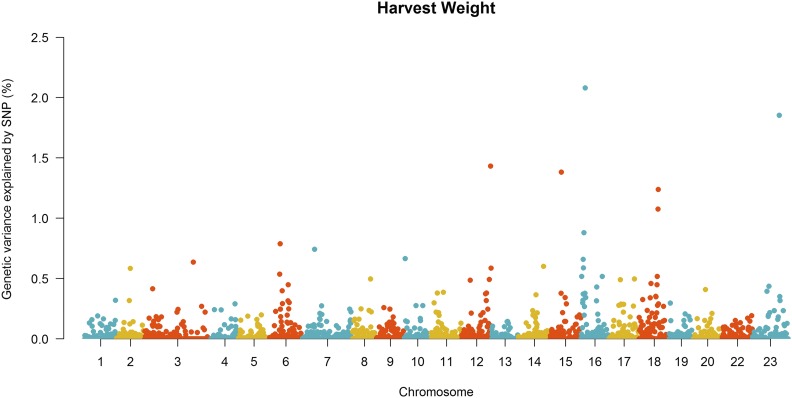
Manhattan plot of genetic variance explained by 20-SNP windows for harvest weight in the 2^nd^ iteration of wssGBLUP.

Manhattan plots for the proportion of genetic variance explained by each 20-SNP window for FY and HW are shown in Figures 1 and 2. A total of 1,624 20-SNP windows with average length of 530 kb (range from 10 to 6,690 kb) were obtained. After the second iteration of wssGBLUP, the top five windows cumulatively explained 5.2 and 8.0% of the total genetic variance for FY and HW, respectively (Table 3).

**Table 3:** Top five ranked 20-SNP windows that explain the largest proportion of genetic variance for fillet yield and harvest weight in Nile tilapia

CHR^1^	Position		Pvar^2^ (%)	Window length (bp)	Genes^3^
	Initial	Final			
**Fillet Yield**					
**LG04**	11,812,441	12,225,401	1.439	412,960	cacng3, clcn7, dctn5
**LG06**	25,191,450	25,573,340	0.981	381,890	adap2, rab11, utp6
LG18*	24,563,797	24,886,884	0.969	323,087	ankrd12, mtcl1, rab31
**LG23**	40,389,783	41,076,465	0.907	686,682	armh1, atp5f1d, fstl3
**LG07**	61,517,871	61,850,135	0.889	332,264	—
**Harvest Weight**					
**LG16**	05,380,219	05,989,612	2.082	609,393	calcrl, gulp1
**LG22**	34,016,287	34,585,708	1.852	569,421	dnai2, ints3, npr1
**LG12**	37,027,113	37,559,043	1.434	531,930	endog, entr1, med27
**LG15**	14,423,480	14,846,999	1.383	423,519	cpsf2, extl3, fut8
LG18*	24,563,797	24,886,884	1.238	323,087	—

* Coincident window for both traits.

1 Chromosome;

2 Percentage of genetic variance explained by each 20-SNP window;

3 *Oreochromis niloticus* used as reference species (full list of genes are available in Table S1).

The full list of genes located within the top five 20-SNPs windows associated with FW and HW is shown in Table S1. Some candidate genes found within the top five most important windows have been suggested to be involved with growth-related traits in previous studies. For FY we identified genes U3 small nucleolar RNA-associated protein 6 homolog (*UTP6*), Ras-related protein (*Rab31*) and Follistatin-related protein (*FLRG* or *FSTL3*), located in chromosome 06, 18 and 23, respectively. For HW we identified the genes Natriuretic Peptide Receptor 1 (*NPR1*) and Exostosin Like Glycosyltransferase 3 (*EXTL3*) located in chromosome 22 and 15, respectively.

### Accuracy of genotype imputation

**Figure 3:**
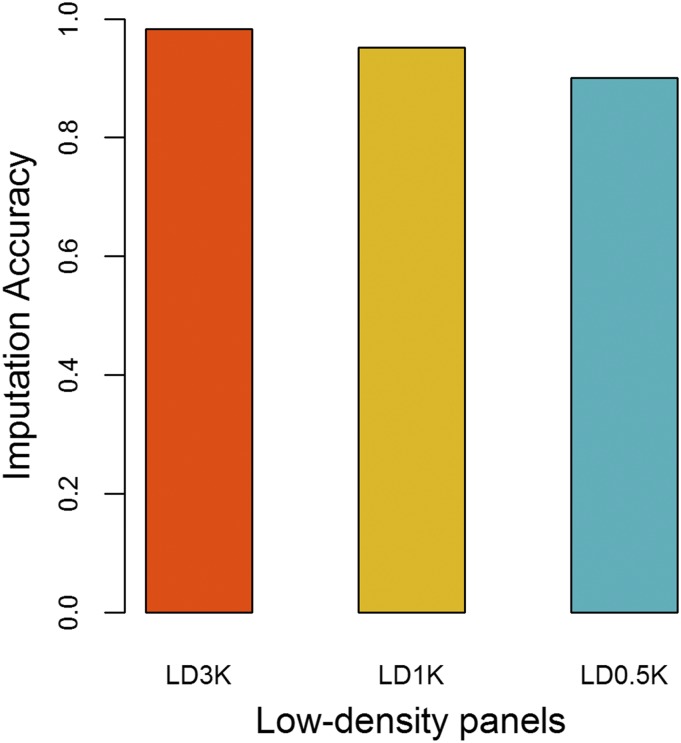
Imputation accuracy from low-density (LD3K, LD1K and LD0.5K) to high-density (HD) panel in Nile tilapia using parents (n = 108) and 20% of offspring (n = 226) genotyped with the HD panel as the reference set and 80% of offspring (n = 904) as the validation set.

We observed that imputation accuracy decreased with reduced marker density going from LD3K to LD0.5K SNP panels with values ranging from 0.98 to 0.90, respectively (Figure 3). The largest increase in imputation accuracy occurred when increasing SNP density from 0.5K to 1K, with an increase in imputation accuracy of about 6%.

Figures S1, S2 and S3 show the correlation between observed and imputed genotypes for each SNP in all chromosomes using the LD3K, LD1K and LD0.5 panels, respectively. Imputation accuracy was not consistent across chromosomes, especially for LD0.5K. Inconsistencies may happen because of the physical position of imputed SNP and the location of the SNP on the LD panel. The imputation accuracy decreased greatly at telomeres, and increased considerably with increased SNP density.

### Accuracy of PBLUP and ssGBLUP

Based on the fivefold cross validation, the prediction accuracy for GEBV from genomic methods outperformed the accuracy for EBV from PBLUP. In addition, the accuracy of genomic selection using imputed genotypes from LD to HD SNP panels outperformed both PBLUP and ssGBLUP using true LD genotypes (Table 4).

**Table 4:** Mean accuracy of EBV and GEBV for fillet yield and harvest weight in Nile tilapia using pedigree-based information, true and imputed genotypes

Traits	Pedigree-based BLUP	True genotypes^1^				Imputed genotypes^2^		
		32K	3K	1K	0.5K	3K	1K	0.5K
**Fillet yield**	0.539	0.621	0.612	0.574	0.560	0.621	0.620	0.585
**Harvest weight**	0.479	0.601	0.539	0.537	0.500	0.607	0.600	0.586

1 High-density (32K) and different true *in silico* low-density panel.

2 Imputed genotypes from different low-density panel (3K, 1K or 0.5K) to high-density panel (32K).

**Figure 4:**
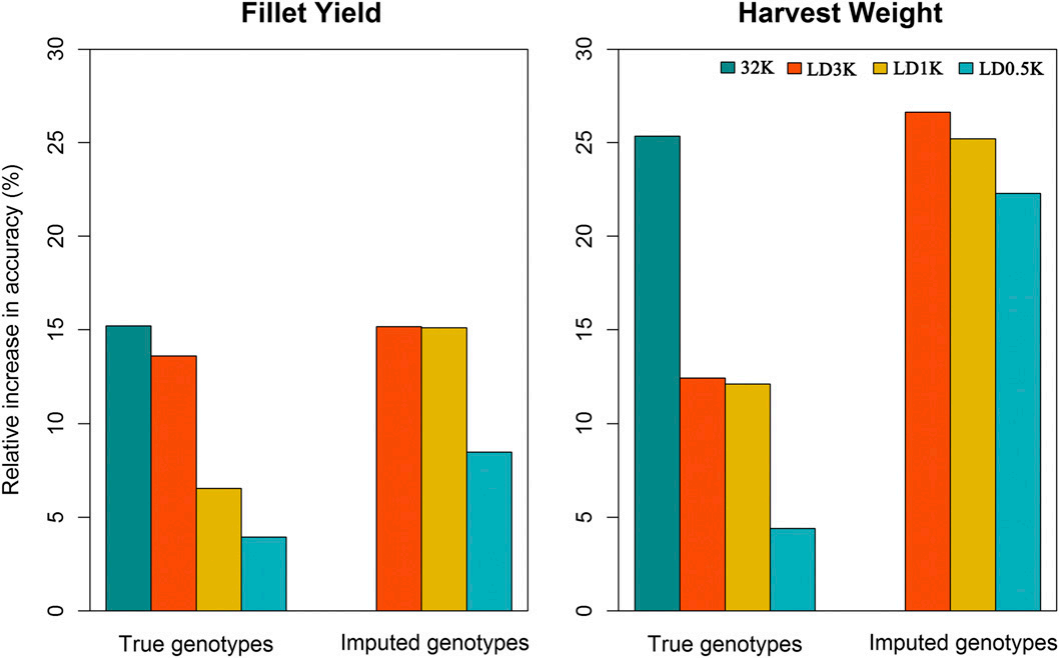
Relative increase in accuracy of different genomic selection methods for fillet yield, harvest weight and waste weight compared to PBLUP in Nile tilapia using true and imputed genotypes.

The relative increase in accuracy of predicted GEBV compared with EBV from PBLUP varied moderately between the LD panels and traits (Figure 4). Thus, the relative increase in accuracy for FY when comparing ssGBLUP to PBLUP ranged from 4 to 15% for true 0.5 and 32K genotypes, respectively and from 8 to 15% for imputed genotypes using the 0.5 and 3K LD SNP panels respectively. For HW the relative increase in accuracy when comparing ssGBLUP to PBLUP ranged from 4 to 25% for true 0.5 and 32K genotypes, respectively and from 22 to 27% for imputed genotypes using the 0.5 and 3K LD SNP panels respectively. In general, the relative increase in accuracy of predicted GEBV from all true LD SNP panels and imputed genotypes were always better than EBV from PBLUP even at the lowest marker density of 0.5 K for all traits. The relative increase in accuracies when comparing ssGBLUP to PBLUP were almost always higher for HW than for FY, except for the use of true 3K genotypes for prediction of FY (14%) which was slightly higher than for HW (13%) using the same 3K genotypes.

The genomic prediction accuracy using imputed genotypes, was identical or very similar between the LD panels compared to the 32K SNP genotypes, especially for FY (Figure 4). When comparing the use of true and imputed genotypes for genomic selection it was evident that genotype imputation resulted in a higher increase in relative accuracy independently of trait and LD SNP panel. As expected, the lowest genomic prediction accuracy when using imputed genotypes was always observed for the SNP panel with lowest imputation accuracy (LD0.5K), which resulted in an accuracy slightly lower than the 32K SNP panel, but higher than PBLUP.

### Cost evaluation

The costs using different LD genotyping panels and different sizes of Nile tilapia breeding populations, ranged from an increase of 4.74% to a reduction of 69.40%, when compared to scenario A, where all animals were genotyped using a HD panel. As expected, scenario D, resulted in the most substantial cost reduction, ranging from 69 to 45%, depending on the size of the breeding population and scenario B resulted in the worst cost reduction (Figure S4). Interestingly, due to value adjustment when increasing the number of samples to genotype, for scenario B and a number of RP + SC equal to 10,150, it is more cost efficient to genotype all animals using a HD panel than genotyping a proportion of animals using LD panel.

## Discussion

### Heritability

We found moderate heritability values for FY and HW which agrees with previous estimates calculated using pedigree-based methods (Gjerde *et al.*, 2012; Nguyen *et al.*, 2010; and Rutten *et al.*, 2005). HW and FY heritability values in tilapia, estimated using genomic information, are reported for the first time in this study. We found slightly higher estimates of heritability when using genomic information compared to PBLUP, especially for HW, which is in accordance with what has been reported in other fish species for fillet yield and growth traits (Tsai *et al.* 2015; Gonzalez-Pena *et al.* 2016).

### Genome-wide association analysis

In the present study, we found no evidence of major quantitative trait loci for both fillet yield and harvest weight in Nile tilapia. The small effect of these loci reinforces evidence of the polygenic nature of these traits. Our results support previous findings which have shown the polygenic nature of fillet yield and growth-related traits in different aquaculture species, with no evidence of major effect genes or genomic regions assessed by GWAS (Gutierrez *et al.* 2015; Tsai *et al.* 2015; Gonzalez-Pena *et al.* 2016; Yoshida *et al.* 2017; Garcia *et al.* 2018; Reis Neto *et al.* 2019). Furthermore, within the five 20-SNP windows that explained the higher proportion of genetic variance, we found several genes that could potentially be involved in growth and fillet yield.

Although, it is out of the scope of the present study to discuss in detail the putative genes involved in FY and HW, we found it worthy to mention some of the most biologically relevant candidates that may be worthy of functional validation. For instance, the *UTP6* gene is suggested to enhance cellular growth through an increase in the number of ribosomes in Chinese hamster ovary cells (Courtes *et al.* 2013). For both FY and HW, between position 24,563,797 and 24,886,884 bp, we identified the *RAB31* gene, which has a role in trafficking the epidermal growth factor receptor (*EGFR*) gene (Chua and Tang 2014), an important receptor of tyrosine kinase in animals that functions in development, growth and tissue regeneration (Wang *et al.* 2018). In addition, *FSTL3*, present in one of the top 5 SNP windows explaining a high proportion of the genetic variance for FY, is a member of follistatin family, which has been suggested to be an inhibitory binding protein of myostatin activity (Hill *et al.* 2002, 2003). Rebhan and Funkenstein (2008) reported experimental evidence that myostatin activity can be inhibited by follistatin. Chu *et al.* (2016) observed an increase in the number of muscle fibers, satellite cell activation and decreased expression of myostatin in animals treated with *FSTL3*; suggesting that the gene might be involved to muscle development in the Chinese Perch (*Siniperca chuatsi*).

In plants *NPR1* is an essential regulator of systemic acquired resistance, conferred immunity to broad-spectrum of pathogens (Cao *et al.* 1997; Mou *et al.* 2003). However, Vanacker *et al.* (2001) reported a novel function for NPR1, which is associated with growth control, cell division and suppressing endoreduplication during leaf development in *Arabidopsis*. In humans, a mutation affected the *EXTL3* gene causing skeletal dysplasia, immune deficiency and development delay. In zebrafish abnormalities of cartilage development and defective formation in fin and branchial arch were reported (Norton *et al.* 2005). Other genes located in a 20-SNP window flanking the top five windows are presented in Table S1.

### Accuracy of genotype imputation

The imputation accuracy, on average, was above 90%, independent of the LD SNP panel used but decreased from LD3K to LD0.5K, which is in accordance with the same pattern seen by Habier *et al.* (2009), Hickey *et al.* (2012) and Yoshida *et al.* (2018b). The imputation errors could be higher in LD panels because they could be less efficient in capturing the linkage and linkage disequilibrium between the markers. Like previous studies we found accuracies of genotype imputation to be very similar using panels of 3K SNPs or denser (Druet *et al.* 2010; Zhang and Druet 2010; Duarte *et al.* 2013; Carvalheiro *et al.* 2014; Cleveland and Hickey 2014; Kijas *et al.* 2016; Tsai *et al.* 2017). However, it is likely that 3K or denser SNP panels will be considerably more expensive than 0.5K or 1K SNP panels, thus cost-effectiveness must be carefully evaluated in further studies.

Some imputation studies tested the size of the reference population (Zhang and Druet 2010; Cleveland and Hickey 2014; Tsai *et al.* 2017; Yoshida *et al.* 2018b), and have shown that the number of animals used in this study should be sufficient to not influence the imputation accuracy. Therefore, we did not include the effect of different genotyping strategies in the final results; however, in preliminary tests we found imputation accuracies lower than 90% using a small proportion of offspring (less than 10%) in the reference set when imputing genotypes from the 0.5K SNP panel (results not show). This is probably because of the small number of animals per family in the reference population, which can influence imputation accuracy (Hickey *et al.* 2012). In this case we had approximately 18 sibs genotyped/family and we used as reference set 20% of offspring in addition to the parents genotyped with the 32K SNP panel to achieve similar accuracy values to those reported by Yoshida *et al.* (2018b) for *Salmo salar*, where 31 sibs/family and just 10% of offspring were needed to surpass an imputation accuracy of 90%.

Figures S1, S2 and S3 indicate regions of the genome containing markers with high imputation errors, especially at the beginning and end of the chromosomes. This is could be an effect of recombination rates, that are known to be higher around the telomeres (Chowdhury *et al.* 2009; Tortereau *et al.* 2012). The physical location of the SNP is another factor that has been shown to be affect the imputation accuracy and to reduce the errors in these regions, some previous studies suggested increasing the coverage of SNP chromosomal extremes (Badke *et al.* 2012; Boichard *et al.* 2012; Dassonneville *et al.* 2012). In addition, high imputation errors far from chromosome extremes can be the result of erratic patterns of linkage disequilibrium, which suggests potential issues related to physical maps and reference genome assembly (Druet *et al.* 2010; Carvalheiro *et al.* 2014; Yoshida *et al.* 2018b).

Another important factor that may affect the imputation accuracy is the linkage disequilibrium between markers (Hickey *et al.* 2012; Carvalheiro *et al.* 2014). A previous study, showed a more rapid decrease of linkage disequilibrium with inter-marker distance for this Nile tilapia population (Yoshida *et al.* 2019a) when compared to other populations of different aquaculture species (Gutierrez *et al.* 2015; Kijas *et al.* 2016; Vallejo *et al.* 2018; Barría *et al.* 2019). Nevertheless, our imputation accuracies are close to the imputation values reported in the literature for salmonids (Kijas *et al.* 2016; Tsai *et al.* 2017; Yoshida *et al.* 2018b) and terrestrial species (Badke *et al.* 2012; Hayes *et al.* 2012; Duarte *et al.* 2013; Hozé *et al.* 2013; Carvalheiro *et al.* 2014), suggesting that the family-based imputation approach is less sensitive to linkage disequilibrium patterns by efficiently exploiting information of highly related animals.

The imputation methods can be classified in family-based and population-based methods. The first exploits linkage information from close related animals and the second uses linkage disequilibrium information of the population (Sargolzaei *et al.* 2014). Previous studies tested the use of different imputation methods as well as different imputation software (Hayes *et al.* 2012; Carvalheiro *et al.* 2014; Sargolzaei *et al.* 2014). Here, we used the FImpute software (Sargolzaei *et al.*, 2014) that consider both family and population-based approaches, or only population-based when the pedigree information is not available. The imputation proceeds using overlapping sliding windows, starting with long haplotypes and moving to short haplotypes (Sargolzaei *et al.*, 2014). This method results in high imputation accuracy when close relatives of targeted individuals are present in the reference group (Carvalheiro *et al.* 2014; Sargolzaei *et al.* 2014; Larmer *et al.* 2014). Furthermore, the computing requirements are considerably lower than other software used for imputation (Sargolzaei *et al.*, 2014).

### Accuracy of genomic prediction

Our results showed that the use of genomic information for estimating breeding values achieved higher accuracies compared to using only pedigree information for FY and HW, independent of the LD SNP panel used, with or without imputation of genotypes (Figure 4). The relative increase in GEBV accuracies compared to PBLUP has been previously reported for growth (Garcia *et al.*, 2018; Tsai *et al.*, 2015) and for different disease resistance traits in farmed aquaculture species (Tsai *et al.* 2016; Vallejo *et al.* 2017; Bangera *et al.* 2017; Correa *et al.* 2017; Barria *et al.* 2018; Yoshida *et al.* 2018a, 2018b).

The accuracy of GEBV depends on some factors such as the number of genotyped and phenotyped individuals in the training population, the heritability and the number of loci affecting the trait (Daetwyler *et al.* 2008; Goddard 2009). Furthermore, the accuracy of genomic prediction is highly dependent on the genotype density used, which means that increasing marker densities tends to generate higher GEBV accuracies (Tsai *et al.* 2016; Bangera *et al.* 2017; Correa *et al.* 2017; Yoshida *et al.* 2018a). In addition, the use of different methods to estimate the GEBV can directly affect the accuracy of genomic prediction. In general, the genomic methods differ in distributional assumptions of marker effects and the calculation of the genetic relationship matrix. Here, we used the ssGBLUP method (Misztal *et al.* 2009), which assumed a normal distribution of marker effects and has some of practical advantages, given that it uses information from genotyped and nongenotyped animals (Lourenco *et al.* 2014), and it has also been demonstrated to provide higher accuracy than the PBLUP method and other genomic methods (Chen *et al.* 2011; Christensen *et al.* 2012; Vallejo *et al.* 2017; Yoshida *et al.* 2018a).

To test the impact of genotype imputation errors in genomic predictions we estimated the accuracy of genomic predictions for FY and HW using imputed genotypic data and compared the data to true 32K and LD SNP genotypes (LD3K, LD1K and LD0.5K). Our results indicate that genomic prediction accuracies using imputed genotypes were always higher than those obtained using true LD genotypes and equal or slightly lower than using true 32K genotypes (Figure 4). For HW the accuracy of GEBV was substantially higher using the imputed LD0.5K than true LD0.5K, LD1K and even LD3K, whereas for FY the accuracy using imputed LD0.5K did not surpass the true LD3K panel. Our results are in accordance with previous studies in aquaculture (Dufflocq *et al.*, 2019; Tsai *et al.*, 2017; Yoshida *et al.*, 2018b) and livestock species (Berry and Kearney 2011; Erbe *et al.* 2012); indicating that the use of genotype imputation can decrease the cost of genotyping by means of using less expensive LD SNP panels without compromising prediction accuracies. In addition, the influence of imputation error on genomic prediction accuracy depends on the genetic architecture underlying the studied traits. For traits that are influenced by few QTL with large effect, accuracy of genomic prediction could be more sensitive to imputation errors than polygenic traits, such FY and HW (Chen *et al.* 2014).

### Implications

The extra costs to genotyping animals represents a key limiting factor for the practical implementation of genomic selection in freshwater fish species. One of the main objectives of this study was to test genotyping imputation as an alternative to reduce the costs of genotyping for genomic selection in Nile tilapia breeding programs. The cost to genotype depends on the density of the SNP panel, genotyping technology used and number of samples. In general, the price ranges from USD $5 to USD $75 for low- (0.5K) and high-density panels (50K), respectively. We did a cost evaluation, that resulted in a reduction of cost ranging from 45 to 69% (Figure S4), assuming different sizes of a breeding population and a strategy aimed to genotyping a proportion of animals using a HD panel and the remaining animals using a 0.5K panel. These values represent the direct savings on genotyping fewer animals using a HD panel and support the results suggested by Dufflocq *et al.* (2019) and Tsai *et al.* (2017) for aquaculture species. The previous authors suggested that the use of imputation strategies can reduce the cost of genotyping by at least 60% compared to genotyping all animals with a HD panel. Therefore, the strategies used in the present study to genotype both parents using a HD panel and a proportion of offspring using a HD and LD panel resulted in accuracies of genomic prediction similar to when all animals were genotyped with the HD panel. These results suggest that genotype imputation can decrease the costs for the practical implementation of genomic prediction in Nile tilapia breeding programs.

## Conclusions

The GWAS indicated a polygenic architecture for fillet yield and harvest weight, with some markers explaining a small proportion of genetic variance; indicating that the implementation of marker assisted selection could not be successfully applied for these traits in the present Nile tilapia population. In contrast, the use of genomic selection could increase the response to selection and improve genetic progress. The use of genotype imputation can reduce genotyping costs and allow the implementation of genomic selection in Nile tilapia breeding programs.
